# Age-related changes in the topological architecture of the brain during hand grip

**DOI:** 10.1016/j.neurobiolaging.2011.08.003

**Published:** 2012-04

**Authors:** Chang-hyun Park, Marie-Hélène Boudrias, Holly Rossiter, Nick S. Ward

**Affiliations:** Sobell Department of Motor Neuroscience and Movement Disorders, Institute of Neurology, University College, London, UK

**Keywords:** Graph-theoretical analysis, fMRI, Hand grip task, Motor performance

## Abstract

Neuroanatomical changes in the aging brain are widely distributed rather than focal. We investigated age-related changes in large-scale functional brain networks by applying graph theory to functional magnetic resonance imaging data acquired during a simple grip task with either dominant or nondominant hand. We measured the effect of age on efficiency of information transfer within a series of hierarchical functional networks composed of the whole brain or component parts of the whole brain. Global efficiency was maintained with aging during dominant hand use, primarily due to increased efficiency in parietal-occipital-cerebellar-related networks. During nondominant hand use, global efficiency, as well as efficiency within ipsilateral hemisphere and between hemispheres declined with age. This was attributable largely to frontal-temporal-limbic-cerebellar-related networks. Increased efficiency with age was seen in networks involving parietal-occipital regions, but unlike for dominant hand use, this topological reconfiguration could not maintain the level of global efficiency. Here, graph theoretical approaches have demonstrated both compensatory and noncompensatory changes in topological configuration of large-scale networks during aging depending on the task.

## 1 Introduction

A range of neurodegenerative and neurochemical changes occur within distributed brain regions with advancing age and are thought to underlie decline in cognitive and motor function (Hackel et al., 1992; Jebsen et al., 1969; Kolb et al., 1998; Potvin et al., 1980). Several studies have attempted to examine the neural correlates of aging using functional imaging and in general, task-related brain activity appears to be focused and lateralized in younger subjects but more diffuse and bilateral in older subjects (Cabeza, 2001). This widespread activity most likely represents an alteration in the way distributed brain regions interact with one another in older subjects, but relatively few studies have explicitly addressed age-related characteristics of large-scale brain networks.

Recently, graph theoretical approaches have been used to measure topological metrics of brain networks. This has allowed the use of neuroimaging to assess large-scale anatomical or functional brain networks in the same way as other complex biological, social, and technological networks. These studies suggest that brain networks have small-world properties, i.e., highly efficient information transfer for a small number of connections across a network (for review see Bassett and Bullmore, 2006). Age-related changes have been seen in brain networks using structural magnetic resonance imaging (MRI) (Zhu et al.), functional MRI (fMRI) (Achard and Bullmore, 2007; Meunier et al., 2009; Wang et al., 2010; Wu et al., 2007), and electroencephalography (Gaál et al., 2010). In general, the effect of age on functional brain networks has been assessed during resting state (Achard and Bullmore, 2007; Gaál et al., 2010; Meunier et al., 2009; Wu et al., 2007) but alterations in task-specific functional brain networks have not yet been thoroughly assessed. One study demonstrated age-related changes in topological patterns of large-scale functional brain networks during memory encoding and recognition (Wang et al., 2010) but such a study has not been performed in the motor domain.

We were therefore interested in looking at age-related changes in large-scale functional brain networks using fMRI data collected during a simple isometric grip task. Previously we have shown that during either simple dominant or nondominant isometric hand grip, magnitude of activity varies with age only in ipsilateral primary motor cortex (M1) (Ward et al., 2008). However, we were interested in whether alterations in network metrics may not be reflected in voxelwise magnitude of activity. Subject-specific measures of network efficiency were calculated for both dominant and nondominant hand grip. We considered hierarchical functional brain networks of different levels from the whole brain to a number of component subregions that constitute the whole brain and looked for age-related correlations in each.

The aims of this study were to investigate the effects of age on the characteristics of functional brain networks used during hand grip and to determine whether a simple alteration in task parameter (switching from dominant to nondominant hand) would alter the age-related changes in topological organization of the brain. Previous studies suggest that motor performance declines more quickly for the nondominant hand. We were interested in whether this would be reflected in functional network characteristics in a way that was not seen using voxelwise metrics (Ward et al., 2008).

## 2 Methods

### 2.1 Subjects

Thirty-two healthy volunteers participated in the study (age range, 22–74 years; mean ± SD, 47.16 ± 15.68 years). These 32 subjects were included in a previous standard general linear model (GLM) analysis to look at age related changes in blood oxygen level-dependent (BOLD) signal (Ward et al., 2008). All subjects were right-handed according to the Edinburgh handedness scale (Oldfield, 1971). They reported no history of neurological illness, psychiatric history, vascular disease, or hypertension. The study was conducted with the understanding and full written consent of each subject and approved by the Joint Ethics Committee of the Institute of Neurology, UCL and National Hospital for Neurology and Neurosurgery, UCL Hospitals NHS Foundation Trust, London.

### 2.2 fMRI data acquisition

Magnetic resonance (MR) images were acquired using a 3T Siemens Allegra system (Siemens AG, Erlangen, Germany). In each Subject T2*-weighted echo-planar MR images were acquired under 2 consecutive sessions of hand grip, 1 using the dominant (right) hand and another using the nondominant (left) hand in a randomized and counterbalanced order across subjects. At each session a total of 120 whole brain images were collected with the BOLD contrast (repetition time = 3120 ms, echo time = 30 ms, number of slices = 48, slice thickness = 2 mm, slice gap = 1 mm, matrix size = 64 × 64, in-plane resolution = 3 mm × 3 mm). A T1-weighted high-resolution sagittal MR image was also acquired for coregistration to echo-planar images in each subject (number of slices = 176, slice thickness = 1 mm, matrix size = 224 × 256, in-plane resolution = 1 mm × 1 mm).

During scanning, subjects performed a series of dynamic isometric hand grips as previously described (Talelli et al., 2008; Ward et al., 2008). A single scanning session comprised 30 visually cued hand grips which included 3 sets of 10 hand grips at each of 15%, 30%, and 45% of each subject's maximum voluntary contraction in a random order. The onset and target force of each single hand grip was visually cued. Subjects practiced hand grip in two 3-minute blocks outside the scanner and then inside the scanner before scanning.

### 2.3 fMRI data preprocessing and statistical analysis

Preprocessing and statistical analysis of imaging data were performed using SPM8 (Wellcome Trust Centre for Neuroimaging, www.fil.ion.ucl.ac.uk/spm/). The first 6 volumes were discarded to allow for T1 equilibration effects, so that 114 volumes were used in preprocessing and statistical analysis. Preprocessing steps included spatial realignment of a series of volumes and unwarping, normalization into the same coordinate frame as the Montreal Neurological Institute (MNI) template brain with transformation parameters derived from segmentation of the high-resolution anatomical image coregistered to the mean functional image, and smoothing using a Gaussian filter of 4 mm full width at half maximum (FWHM).

Statistical analysis of preprocessed functional images was performed for the dominant and nondominant hand sessions separately. In each, hand grips were modeled as delta functions together with a second mean corrected covariate comprising the delta function scaled by the actual peak force exerted for each hand grip. Both covariates were convolved with a canonical synthetic hemodynamic response function and used in a general linear model (Friston et al., 1994) together with a single covariate representing the mean (constant) term over scans.

### 2.4 Graph-theoretical analysis

Ninety-three cortical and subcortical areas over the whole brain were defined based on the automated anatomical labeling brain atlas (Tzourio-Mazoyer et al., 2002). The complete list of 93 brain areas is shown in Table 1. For each brain area, voxelwise BOLD signal time series were extracted from 114 preprocessed scans, whitened, and high pass filtered at 128 Hz. Each time series was adjusted to include only effects of hand grip by removing the part explained by covariates other than the hand grip covariate because we were interested in age-related effects during hand grip, irrespective of how forcefully subjects actually performed hand grip.

The representative time series was taken as the first eigenvariate from the singular value decomposition of a matrix composed of each time series from all voxels within the brain area. Finally, 93 representative time series, each with 114 values, were collected and a 93 × 93 matrix {*r*_ij_} of Pearson's correlation coefficients between all pairs of representative time series was calculated.

**Fig. 1:**
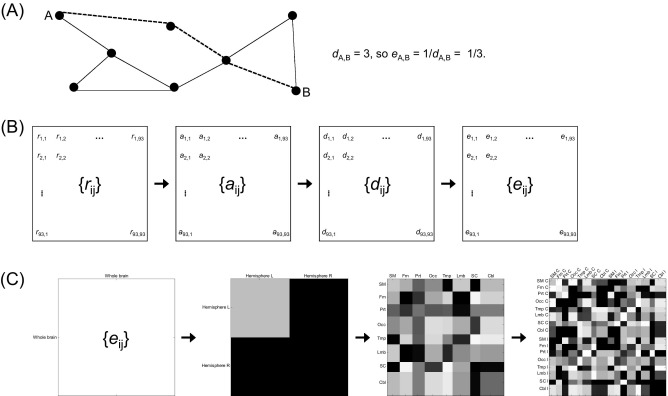
(A) Schematic explanation of measuring the shortest path length and efficiency between a pair of areas in a network, (B) a procedure for acquiring a matrix {*e*_ij_} of the whole brain network efficiency, and (C) submatrices of {*e*_ij_} for measuring efficiencies of the component networks. In (A), between A and B in an example network, the shortest path length *d*_A,B_ is 3 from the minimum number of connections across the path displayed as a dotted line and the efficiency *e*_A,B_ is 1/3 as the reciprocal of *d*_A,B_. In (B), {*r*_ij_}, {*a*_ij_} and {*d*_ij_} represent a correlation coefficient matrix, an adjacency matrix, and a shortest path length matrix for the whole brain network respectively. In (C), representations of functional brain networks on {*e*_ij_} are for the whole brain network, hemispheric networks, brain regional networks for 8 brain regions, and brain regional networks for 16 brain regions, in order from left to right. Abbreviations: C, contralateral; Cbl, cerebellar; Frn, frontal; I, ipsilateral; Lmb, limbic and paralimbic; Occ, occipital; Prt, parietal; SC, subcortical; SM, sensorimotor; Tmp, temporal.

For the whole brain network, a matrix {*r*_ij_} of Pearson's correlation coefficients was converted into an adjacency matrix {*a*_ij_} consisting of 1's and 0's by applying a correlation coefficient threshold value to {*r*_ij_}. In {*a*_ij_}, 1's indicate functional connections and 0's indicate no functional connections between 2 brain areas. A matrix {*d*_ij_} of the shortest path length between each of the 93 brain areas was acquired from the adjacency matrix {*a*_ij_} (Fig. 1A). In this study we used only undirected and unweighted functional brain networks, so the shortest path length *d*_ij_ is just the minimum number of functional connections between 2 brain areas *i* and *j*. Our metric of interest, efficiency *e*_ij_ between 2 brain areas *i* and *j*, was defined to be inversely proportional to the shortest path length *d*_ij_:*e*_ij_ = 1/*d*_ij_ (Latora and Marchiori, 2001) and so a matrix {*e*_ij_} of the efficiency of connections between each of the 93 brain areas was calculated as the reciprocal of every element of {*d*_ij_} (Fig. 1B).

Having calculated the matrix of efficiency {*e*_ij_}, we considered efficiency of connections within each of the following series of hierarchical component functional networks: (1) whole brain comprising all 93 brain areas; (2) within contralateral (to hand movement) hemisphere, within ipsilateral hemisphere, and between hemispheres (each hemisphere comprising 46 brain areas after excluding the midline cerebellar vermis); (3) within and between each of 8 component regions—defined as bilateral sensorimotor, frontal, parietal, occipital, temporal, limbic and paralimbic, subcortical, and cerebellar brain regions (where a brain region comprises several component brain areas—see Table 1); and (4) within and between each of 16 component regions—defined as contralateral or ipsilateral sensorimotor, frontal, parietal, occipital, temporal, limbic and paralimbic, subcortical, and cerebellar brain regions.

The efficiency *E* of the whole brain network was calculated by normalizing the sum of every element of {*e*_ij_} by its maximal possible value for complete connections so that 0 ≤ *E* ≤ 1. The efficiency of a component network was calculated by normalizing the sum of the elements of a submatrix of {*e*_ij_} corresponding to the component network by its maximal possible value. The reason we calculated the efficiency of a component network using a subspace of {*e*_ij_} is that we searched for a contribution of each component network toward the efficiency of the whole brain network not by regarding the component network as independent and isolated. Representations of component networks of {*e*_ij_} are displayed in Fig. 1C.

When converting {*r*_ij_} into {*a*_ij_}, different correlation coefficient threshold values generated whole brain networks of different numbers of functional connections, or connection density which was determined as the ratio of the number of existing functional connections to the number of all possible functional connections between every pair of brain areas. For a specific connection density, an individual correlation coefficient threshold value was applied to each subject's {*r*_ij_} so that {*a*_ij_} has the same number of functional connections across subjects.

To avoid sticking to an arbitrary choice of connection density, we explored different connection densities across a range from 0.10 to 0.40 with an increment of 0.05 for which small-world properties were preserved. Lower and higher connection densities outside the range were not considered for avoiding the occurrence of isolated connections in networks at lower connection densities and keeping sparse networks which could be failed at higher connection densities even though small-world properties were satisfied at those connection densities. Small-world properties of the whole brain network were assessed by computing small-worldness values, *E*_loc_/*E*_loc,rand_ and (*E*_loc_/*E*_loc,rand_)/(*E*_glob_/*E*_glob,rand_), compatible with those previously described (Humphries et al., 2006). *E*_glob_ and *E*_loc_ are global efficiency and local efficiency of the whole brain network respectively, as initially introduced in network studies (Latora and Marchiori, 2001). *E*_glob_ of the whole brain network is just the efficiency *E* of the whole brain network described above. *E*_glob,rand_ and *E*_loc,rand_ are global efficiency and local efficiency of the comparable random network that has the same number of connections as the whole brain network. At each connection density, we randomly generated the comparable random network 100 times and then measured *E*_glob,rand_ and *E*_loc,rand_ as averages. Both the small-worldness values *E*_loc_/*E*_loc,rand_ and (*E*_loc_/*E*_loc,rand_)/(*E*_glob_/*E*_glob,rand_) higher than 1 can be regarded as showing small-world properties of the whole brain network.

Having calculated the matrix of efficiency {*e*_ij_} for each subject, we examined which connections varied with age, by calculating Pearson's correlation coefficient between subjects' network efficiencies and ages, and assessed the statistical significance of the age correlation at a false discovery rate corrected *p* value 0.05. Also, we assessed statistical significance of the difference between 2 Pearson's correlation coefficients corresponding to dominant and nondominant hand grip at a false discovery rate corrected *p* value 0.05 for the functional brain network comprising the same brain areas.

## 3 Results

### 3.1 Small-world properties of the whole brain network

**Fig. 2:**
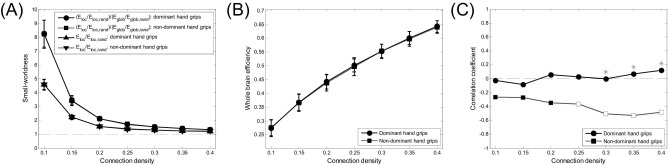
(A) Small-worldness values *E*_loc_/*E*_loc,rand_ and (*E*_loc_/*E*_loc,rand_)/(*E*_glob_/*E*_glob,rand_) of the whole brain network, (B) efficiency of the whole brain network, and (C) correlation between whole brain network efficiency and age. All measures are represented as functions of connection density across a range from 0.10 to 0.40 with an increment of 0.05. Circles and squares in (A) and (B) and upper triangles and downward triangles in (A) represent means and error bars in (A) and (B) represent standard deviations across all subjects. In (C), empty squares indicate significant correlation coefficients and stars indicate significant differences in correlation between dominant and nondominant hand grip.

The small-world topology changes with connection density. Fig. 2A shows small-worldness values of the whole brain network as a function of connection density. Across a range of connection densities from 0.10 to 0.40, small-worldness values *E*_loc_/*E*_loc,rand_ and (*E*_loc_/*E*_loc,rand_)/(*E*_glob_/*E*_glob,rand_) of the whole brain network are higher than 1, so that small-world properties are considered to be preserved in the range of connection densities.

### 3.2 Changes in whole brain network efficiency with age

Fig. 2B shows the efficiency of the whole brain network as a function of connection density and Fig. 2C shows correlation coefficients between the whole brain network efficiency and age as a function of connection density. Although the whole brain network efficiency is not significantly different between dominant and nondominant hand grip at any connection density, the correlation between age and whole brain network efficiency is significantly negative only for nondominant hand grip at network densities of 0.25 to 0.4, and moreover, correlation coefficients are significantly different between dominant and nondominant hand grip at network densities of 0.3 to 0.4.

### 3.3 Changes in intra- and interhemispheric network efficiency with age

**Fig. 3:**
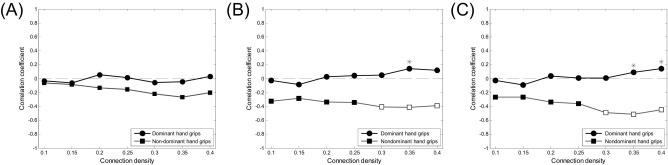
Correlation between hemispheric network efficiency and age, specifically for (A) contralateral hemispheric, (B) ipsilateral hemispheric, and (C) interhemispheric networks. Correlation coefficients are represented as functions of connection density across a range from 0.10 to 0.40 with an increment of 0.05. Empty squares indicate significant correlations and stars indicate significant differences in correlation between dominant and nondominant hand grip.

Correlation coefficients between the contralateral (left for dominant hand grip and right for nondominant hand grip) and ipsilateral (right for dominant hand grip and left for nondominant hand grip) hemispheric network efficiency and age are shown in Fig. 3A and B. The correlation between efficiency of both hemispheric networks and age is not significant for the dominant hand. However, the correlation of the ipsilateral hemispheric network efficiency and age is significantly negative for nondominant hand grip at connection densities of 0.3 to 0.4 and correlation coefficients are significantly different for the dominant and nondominant hands at a connection density of 0.35.

The correlation between interhemispheric network efficiency and age (Fig. 3C) is significantly negative for the nondominant hand at connection densities of 0.3 to 0.4 and correlation coefficients are significantly different for the dominant and nondominant hands at connection densities of 0.35 to 0.4.

### 3.4 Changes in brain regional network efficiency with age

**Supplementary Fig. 1:**
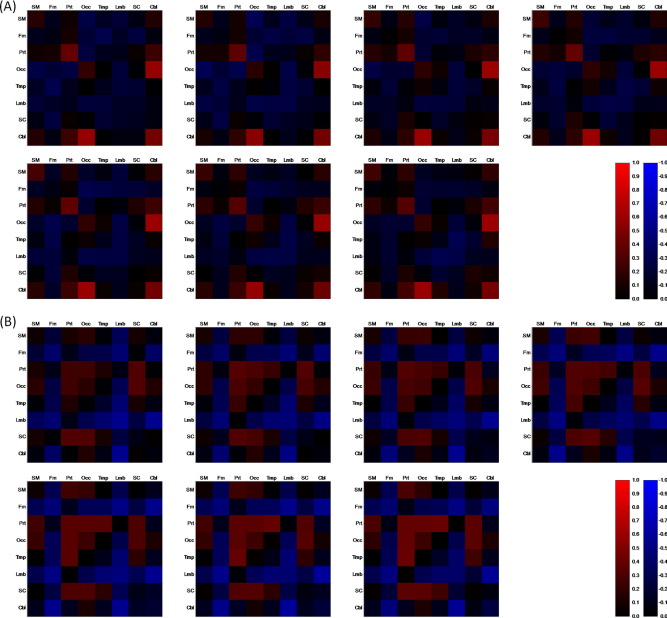
Distribution of correlation coefficients between brain regional efficiency and age at connection densities from 0.10 to 0.40 with an increment of 0.05 for (A) dominant and (B) nondominant hand grip when considering 8 brain regions. Black-red and black-blue indicate positive and negative correlations respectively between age and efficiency of the corresponding intra- or interregional networks. Abbreviations: Cbl, cerebellar; Frn, frontal; Lmb, limbic and/or paralimbic; Occ, occipital; Prt, parietal; SC, subcortical; SM, sensorimotor; Tmp, temporal.

**Supplementary Fig. 2:**
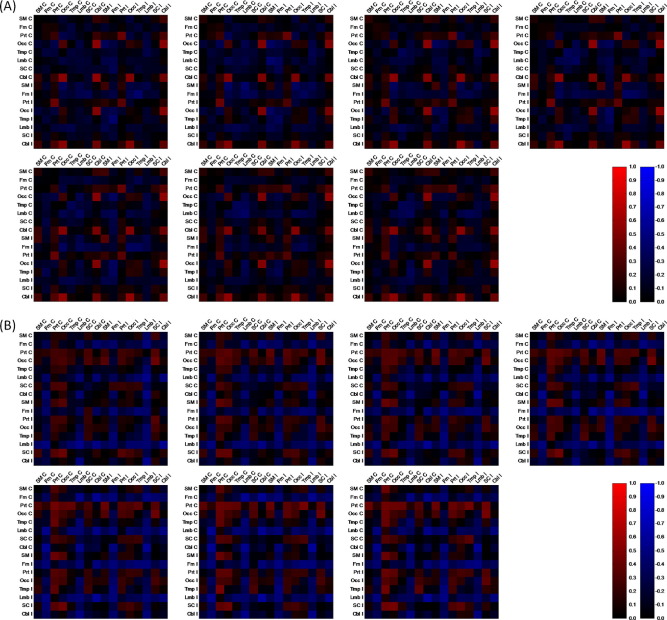
Distribution of correlation coefficients between brain regional efficiency and age at connection densities from 0.10 to 0.40 with an increment of 0.05 for (A) dominant and (B) nondominant hand grip when considering 16 brain regions. Black-red and black-blue indicate positive and negative correlations respectively between age and efficiency of the corresponding intra- or interregional networks. Abbreviations: C, contralateral; Cbl, cerebellar; Frn, frontal; I, ipsilateral; Lmb, limbic and/or paralimbic; Occ, occipital; Prt, parietal; SC, subcortical; SM, sensorimotor; Tmp, temporal.

**Fig. 4:**
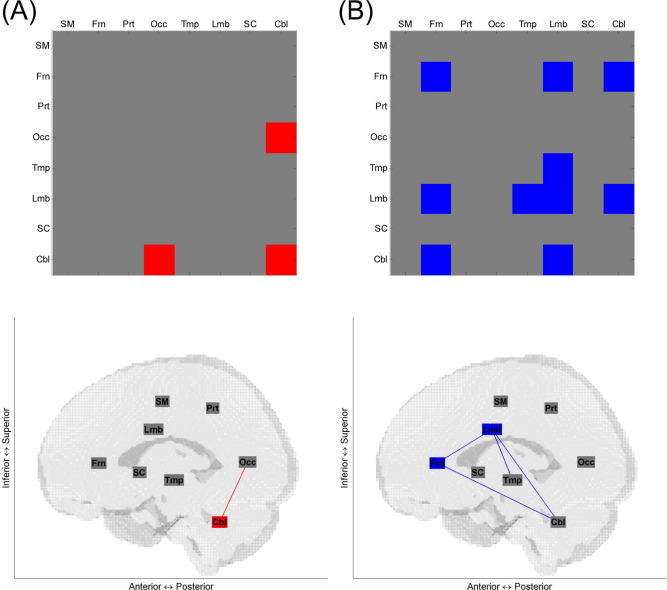
Brain regional networks that showed significant age-efficiency correlations across more than half of the range from 0.10 to 0.40 of connection densities for (A) dominant and (B) nondominant hand grip when considering 8 brain regions. In the upper and lower panels, red and blue indicate positive and negative correlations respectively between age and efficiency of the corresponding intra- or interregional networks. In the lower panel, boxes are used for intraregional network efficiency and lines for interregional network efficiency. Abbreviations: Cbl, cerebellar; Frn, frontal; Lmb, limbic and paralimbic; Occ, occipital; Prt, parietal; SC, subcortical; SM, sensorimotor; Tmp, temporal.

**Fig. 5:**
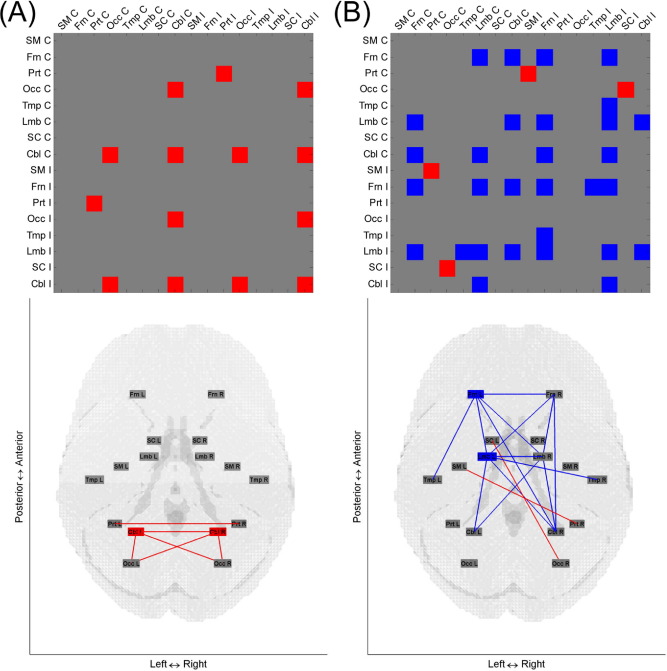
Brain regional networks that showed significant age-efficiency correlations across more than half of the range from 0.10 to 0.40 of connection densities for (A) dominant and (B) nondominant hand grip when considering 16 brain regions. In the upper and lower panels, red and blue indicate positive and negative correlations respectively between age and efficiency of the corresponding intra- or interregional networks. In the lower panel, boxes are used for intraregional network efficiency and lines for interregional network efficiency. Abbreviations: C, contralateral; Cbl, cerebellar; Frn, frontal; I, ipsilateral; L, left; Lmb, limbic and paralimbic; Occ, occipital; Prt, parietal; R, right; SC, subcortical; SM, sensorimotor; Tmp, temporal.

Fig. 4 shows connections within and between the 8 brain regions for which brain regional network efficiency and age correlated in over half of the considered range of connection densities for dominant and nondominant hand grip. Fig. 5 shows results when 16 brain regions (separately for contralateral and ipsilateral hemispheres) were considered using the same criteria. The matrices of age-efficiency correlation without thresholding at every connection density are shown in Supplementary Fig. 1 for 8 brain regions and in Supplementary Fig. 2 for 16 brain regions. When comparing brain regional networks that showed significant correlation between age and efficiency across more than half of the considered range of connection densities, no brain regional networks overlapped between dominant and nondominant hand grip.

For dominant hand grip, positive correlations between age and efficiency were found in parietal-occipital-cerebellar-related networks when considering both 8 and 16 brain regions (Figs. 4A and 5A). There were no significant negative correlations between age and efficiency when using the dominant hand.

For nondominant hand grip, positive correlations between age and efficiency were found only when considering 16 brain regions, in left sensorimotor-right parietal and right occipital-left subcortical networks (Fig. 5B). There were negative correlations between age and efficiency in frontal-temporal-limbic-cerebellar-related networks when considering both 8 and 16 brain regions (Figs. 4B and 5B).

**Supplementary Fig. 3:**
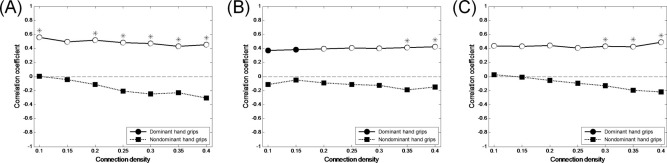
Correlation between cerebellar network efficiency and age, specifically for (A) contralateral cerebellar, (B) ipsilateral cerebellar, and (C) intercerebellar networks. Correlation coefficients are represented as functions of connection density across a range from 0.10 to 0.40 with an increment of 0.05. Empty circles indicate significant correlation coefficients and stars indicate significant differences in correlation between dominant and nondominant hand grip.

**Fig. 6:**
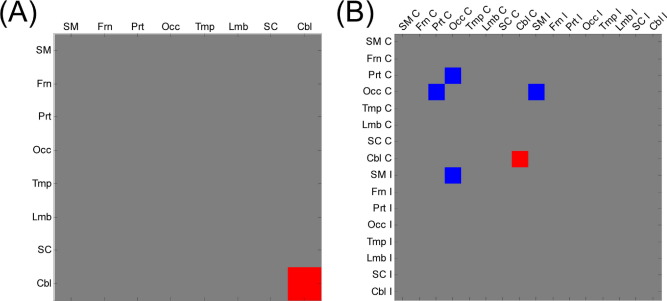
Brain regional networks that showed significant differences in age-efficiency correlation between dominant and nondominant hand grip across more than half of the range from 0.10 to 0.40 of connection densities when considering (A) 8 brain regions and (B) 16 brain regions. Red and blue indicate higher and lower age-efficiency correlations respectively for dominant hand grip compared with nondominant hand grip. Abbreviations: C, contralateral; Cbl, cerebellar; Frn, frontal; I, ipsilateral; Lmb, limbic and paralimbic; Occ, occipital; Prt, parietal; SC, subcortical; SM, sensorimotor; Tmp, temporal.

A comparison of age-efficiency correlations between dominant and nondominant hands revealed significant differences in the cerebellar network. Fig. 6 shows connections of brain regions for which age-efficiency correlations were different between dominant and nondominant hands in over half of the considered range of connection densities when 8 and 16 brain regions were considered. The cerebellar network, particularly the contralateral cerebellar network, displays a significant difference. Age-efficiency correlations in the ipsilateral cerebellar and intercerebellar networks as well as the contralateral cerebellar network are significantly positive only for dominant hand grip, as shown in Supplementary Fig. 3.

## 4 Discussion

In this study we examined the characteristics of functional brain networks during hand grip and how they were affected by age and hand dominance. We analyzed fMRI data using graph theory to show that the efficiency of the whole brain and widely distributed component networks employed during the performance of a simple motor task was related to age. Secondly, we have shown that the nature of these correlations varied depending on whether the dominant or nondominant hand was used. Previous fMRI studies using more complex motor tasks have demonstrated increased task-related brain activity in widely distributed, often nonmotor, brain regions with advancing age (Goble et al., 2010; Heuninckx et al., 2005; Hutchinson et al., 2002; Van Impe et al., 2009), but here we used a simple isometric hand grip task with visual feedback. Previously we measured the voxelwise magnitude of brain activity during this same hand grip task and found age related variations only in ipsilateral primary motor cortex, whether using the dominant or nondominant hand (Ward et al., 2008). This finding was subsequently attributed to diminished interhemispheric inhibition from contralateral to ipsilateral primary motor cortex during hand grip with age (Talelli et al., 2008). However, neuroanatomical changes in the aging brain are widely distributed rather than focal and so here we were interested in whether more generalized age-related changes were evident in the efficiency of much broader functional brain networks during the same simple hand grip task. In order to address this question, we applied graph theory to the functional imaging data. The differences in the topological distribution of the results suggest that analysis of these data using graph theory appears able to detect subtle interactions within brain networks which cannot be captured by examining localized voxelwise brain activity in isolation.

Previous work has shown that the efficiency of the whole brain network (global efficiency) or reciprocal shortest path length diminishes with increasing age. This has been assessed in anatomical networks (Zhu et al., 2010), resting state functional networks (Achard and Bullmore, 2007), and during cognitive performance (Wang et al., 2010). In the current study the properties of functional brain networks were assessed during simple motor performance with either dominant or nondominant hand. Overall, there were no differences in global efficiency between hands when averaging across the whole group, but we found that global efficiency diminished with age only when the nondominant hand was used (Fig. 2C) in keeping with studies suggesting age-related differences in brain activity are more prominent when the nondominant rather than the dominant hand is used (Hutchinson et al., 2002).

To investigate this effect of age on brain network efficiency in more detail we examined a series of component networks. The efficiency of a component network was calculated by summing the efficiencies within the component network and then dividing by the maximum possible value. When considering the hemispheres separately, we found that efficiency was significantly decreased in both the ipsilateral hemispheric and interhemispheric (all left-to-right and right-to-left connections) networks for nondominant but not for dominant hand grip with advancing age (Fig. 3). In other words reduced global efficiency during nondominant hand use in older subjects was attributable to impaired information transfer predominantly within the ipsilateral hemisphere as well as between hemispheres, but not within the contralateral hemisphere. Age-related reduction in hemispheric lateralization has been shown in sensorimotor regions (for motor tasks) (Mattay et al., 2002; McGregor et al., 2009; Naccarato et al., 2006; Ward and Frackowiak, 2003; Ward et al., 2008) and in frontal regions (for cognitive tasks) (for review refer to Rajah and D'Esposito, 2005). These results have largely been due to more bilateral task-related brain activity, often interpreted as compensatory activity. However, another interpretation is that this increase in activity is due to a reduced ability to modulate inhibitory processes (Fujiyama et al., 2009). It is interesting that our current results demonstrate reduced ipsilateral hemisphere efficiency with aging. If such a finding were seen consistently across a number of different tasks it would suggest that an increase in task-related ipsilateral brain activity in the context of aging might reflect reduced rather than increased efficiency of information transfer. The quantitative relationship between the magnitude of regional activity and network efficiency would be interesting to explore in future studies.

It might be interesting that the reduced hemispheric network efficiency with aging was observed in the left hemisphere during left hand use while the left hemisphere would be considered to be dominant in right-handers. However, it is worth distinguishing between dominance in terms of handedness and dominance with respect to use of a specific hand in hemispheres. Even in right-handers, the left hemisphere would be considered to be nondominantly involved during left hand use. Thus, the reduced hemispheric network efficiency in the left hemisphere during left hand use could be rephrased as the reduced hemispheric network efficiency in the nondominantly involved hemisphere during nondominant hand use.

Efficiency of the whole brain and component hemispheric networks during grasping with the dominant hand did not alter with age. However, during the same task, efficiency of parietal-occipital-cerebellar-related networks increased with age. This might represent a form of configurational compensation which maintains overall global and hemispheric efficiency. Conversely, when using the nondominant hand, a decline of whole brain network efficiency was seen. This can be accounted for by decreased efficiency within the ipsilateral hemisphere with age, specifically the frontal-temporal-limbic-cerebellar-related networks. Increased efficiency with age was seen in connections between left sensorimotor and right parietal regions as well as between right occipital and left subcortical regions. If this increase in efficiency represented an attempt at configurational compensation, then it was unsuccessful as high global efficiency was not maintained as in the level of younger subjects with aging.

The cerebellar region was involved in both increased efficiency with age for dominant hand grip and decreased efficiency with age for nondominant hand grip. Furthermore, age-efficiency correlations in the cerebellar region were different between dominant and nondominant hand grip (Fig. 6), so it is proposed that the cerebellar region may play a key role in determining the success or failure of configurational compensation.

In our study, the task was a simple one which subjects did not find more difficult to perform with either hand. There was no correlation between maximum grip strength with either hand and age, but it is possible that the older subjects performed the task differently to the younger ones and perhaps this difference was exaggerated when the nondominant hand was used. Motor performance declines with age, probably more so in tasks requiring fine manipulation, and when using the nondominant hand (Smith et al., 1999). The failure of topological reconfiguration to maintain levels of global efficiency may be the cause of steeper age-related decline in motor performance during nondominant hand use.

A potential weakness of this study is that we do not have a behavioral correlate to suggest relatively worse performance of the grip task with the nondominant hand; age related decline in motor performance is more readily seen in more complex tasks (Smith et al., 1999). However, this would have been difficult because a simple task was chosen in order to equate the level of performance across subjects and therefore avoid confounding differences in performance. In future, the relevance of configurational compensation to functional compensation, i.e., changes that support successful performance by counteracting age-related declines in brain function, will need to be addressed explicitly by manipulating performance level within subjects.

In the results of this study, significant correlations between age and efficiency tend to occur at higher connection densities. Without any practical way to select a specific threshold value that gives true connections, consideration of a range of connection densities is common when using graph theoretical approaches. The selected range of connection densities can be considered as exploratory and hypothetical, so that consistent statistical significance across the whole range of connection densities may not be expected. On the other hand, the tendency of statistical significance to be shown at higher connection densities might be partially attributable to the fluctuation of network metric values in networks including isolated connections which are often found at lower connection densities.

In this study, by considering hierarchical brain networks, we found the contribution of each component network at different levels toward correlation of the efficiency with age at the whole brain level. We focused on revealing the hierarchical relationship between brain networks at different levels, rather than explaining functional relevance of specific brain regions or networks. “Configurational compensation” has been proposed as the concept describing the hierarchical relationship which affected task parameter-dependent age correlations for the whole brain network.

In spite of novel approaches to functional brain networks, there are some limitations in this study. We used Pearson's correlation as an association measure for constructing an association matrix. Partial correlation was not used because the whole brain network constructed with it did not satisfy small-world properties, despite its high sensitivity to network connection detection (Smith et al., 2011). Moreover, even after constructing an association matrix, influences of the selection of threshold values to convert an association matrix into an adjacency matrix on topological metrics may be a drawback of graph-theoretical approaches in general. The adequacy of choices of an association measure and a range of plausible connection densities derived by the selection of threshold values needs to be validated further and ultimately methods for discovering a network that explains observed time series best (Friston et al., 2011) would be demanded, especially with respect to the experimental question in task-driven fMRI data as in this study.

Graph-theoretical approaches have often been applied to data collected in the resting state. Here, however, we were interested in the characteristics of brain networks during the performance of 2 similar motor tasks. Our results suggest that network characteristics assessed during a task are very different from those measured at rest, but also that they are sensitive to minor changes in the task itself. Although motor tasks primarily involve activity in the sensorimotor system, more widespread brain areas are clearly involved and this is shown even more clearly by our graph theory network approach. Moreover, consideration of hierarchical brain networks can be useful for finding changes in smaller components of the whole brain in order to address questions about topological configurational compensation. In future, findings in component networks could be related to other methods of assessing the functional and effective connectivity of brain networks, in order to derive a more integrated view of brain reorganization in health and disease.

## Disclosure statement

All authors disclose no conflicts of interest.

Appropriate approval and procedures were used concerning human subjects in this research.

## Figures and Tables

**Table 1:** Brain areas used for graph-theoretical analysis

Brain region	Number	Brain area
Sensorimotor (SM)	1/2	Precentral gyrus
	3/4	Rolandic operculum
	5/6	Supplementary motor area
	7/8	Postcentral gyrus
	9/10	Paracentral lobule
Frontal (Frn)	11/12	Superior frontal gyrus
	13/14	Middle frontal gyrus
	15/16	Inferior frontal gyrus
	17/18	Olfactory cortex
	19/20	Gyrus rectus
Parietal (Prt)	21/22	Superior parietal gyrus
	23/24	Inferior parietal gyrus
	25/26	Supramarginal gyrus
	27/28	Angular gyrus
	29/30	Precuneus
Occipital (Occ)	31/32	Calcarine fissure and surrounding cortex
	33/34	Cuneus
	35/36	Lingual gyrus
	37/38	Superior occipital gyrus
	39/40	Middle occipital gyrus
	41/42	Inferior occipital gyrus
	43/44	Fusiform gyrus
Temporal (Tmp)	45/46	Heschl gyrus
	47/48	Superior temporal gyrus
	49/50	Middle temporal gyrus
	51/52	Inferior temporal gyrus
Limbic and paralimbic (Lmb)	53/54	Insula
	55/56	Anterior cingulate gyrus
	57/58	Median cingulate gyrus
	59/60	Posterior cingulate gyrus
	61/62	Hippocampus
	63/64	Parahippocampal gyrus
	65/66	Amygdala
Subcortical (SC)	67/68	Caudate nucleus
	69/70	Putamen
	71/72	Globus pallidus
	73/74	Thalamus
Cerebellar (Cbl)	75/76	Cerebellar hemispheric lobule crus 1
	77/78	Cerebellar hemispheric lobule crus 2
	79/80	Cerebellar hemispheric lobule 3
	81/82	Cerebellar hemispheric lobule 4–5
	83/84	Cerebellar hemispheric lobule 6
	85/86	Cerebellar hemispheric lobule 7b
	87/88	Cerebellar hemispheric lobule 8
	89/90	Cerebellar hemispheric lobule 9
	91/92	Cerebellar hemispheric lobule 10
	93	Cerebellar vermis

A total of 93 brain areas, including left and right areas except for the cerebellar vermis composed the whole brain network.
